# The Varioloid Disease

**Published:** 1838-09-26

**Authors:** N. Chapman

**Affiliations:** Professor of the Theory and Practice of Physic, in the University of Pennsylvania


### THE MEDICAL EXAMINER.

DEVOTED TO MEDICINE, SURGERY, AND THE COLLATERAL SCIENCES.

EDITED BY W. W. GERHARD, M.D., J. B. BIDDLE, M.D., AND M. CLYMER, M.D.

No. 20.] PHILADELPHIA, WEDNESDAY, SEPTEMBER 26, 1838. [Vol. I.

### THE VARIOLOID DISEASE.

*By* N. Chapman, M. D., *Professor of the Theory and Practice of Physic, in the University of Pennsylvania.*

(Continued from page 299.)

Connected with this inquiry, the facts are now, perhaps, sufficiently laid open to enable me to enter on those speculations, which immediately grow out of the subject. The question primarily arising relates to the precise nature of the epidemic, whether it really be small-pox, or some other affection not obedient to exactly the same laws.

Two hypotheses have been advanced, each of which is maintained with much ingenuity, and force of argument. The predominant opinion in Europe, as well as in this country, supposes it to be genuine small-pox, which, operating on a system that has undergone the variolous or vaccine impression, produces a comparatively mild affection, to which the title modified small-pox, or Varioloid Disease, is applied.

In support of this view, it is alleged, that the most lenient and worst forms of the epidemic, in some instances, reciprocally produce each other, either by inoculation, or in the natural way, and this is held forth as the *experimentum crucis*, from which there is no escape. But, it seems to me, to be rather a *petitio principii*, in which the variolous character of the case is assumed, and not demonstrated. The fact of their mutual communicability, admitting it to be true, which I am not disposed to do, as regards the disease particularly among us, surely does not prove the identity with small-pox, since another disease, in its different states, may have the same property of reciprocity of production. Never have I known the varioloid miasm to induce variola,—nor is there any positive evidence within my own experience, of its being infectious, causing a disease even like itself. That a contrary opinion exists, I am aware, though I think it has been hastily formed, and is not entitled to much respect. Nor is it readily imparted by inoculation, and never, so far as I have seen, was small-pox, or any near approach to it, the result of the operation. Most of the attempts to propagate it in this mode, have utterly failed. Experiments, however, made here in 1823, show, that the varioloid virus introduced into a system, neither variolated nor vaccinated previously, occasions a vaccine-like vesicle, around which are soon thrown a number of pimples which run into each other, and into the vesicle, after which, the latter degenerates into an irregular phlegmon, followed by fever, and a slight eruption: whereas, on a protected system, only a local vesicle is induced, resembling the vaccine, which perishes prematurely on the sixth day. These experiments, which were originally made by Dr. Darragh of this city, have since been confirmed on repetition by M. Genderin of Paris, who even goes further, and says, that the same protective effect is derived from the varioloid virus in this way as by variolation, or vaccination.

Whatever may have been the disease elsewhere, it is hardly possible to conceive, that as it existed at Edinburgh, and especially in this city, it could have been pure small-pox. There are several discrepancies hardly reconcilable with such a supposition. Not to dwell on minor points of difference, it seized indiscriminately, though not in an equal degree, on the vaccinated, the variolated, and unprotected, by either of these processes, and in some instances, there were two or three reiterations of attack in the same person, within a very short time,—circumstances unprecedented in the history of small-pox!

Exigetically, it is suggested, that at all times, such occurrences were more common in regard to small-pox than suspected, and especially during an epidemic prevalence of it. There is, perhaps, some foundation for this allegation. The conviction was very general among the early writers on the disease, that it might be repeated in the same person. This doctrine was indeed asserted, with few exceptions, till arraigned by Mead in a very confident tone, by whose weight of authority it came to be subverted, or, at all events, very much shakened. Cases militating against his views, were henceforward, till lately, denied, for the most part, to be of a variolous nature, and explained away as some other distinct eruption. But the ancient notion has again been revived, and it is now maintained, that the eruptions called chicken-pock, swine-pock, horn-pock, stone-pock, water-pock, crystalline-pock, are really the variolous disease, thus variously modified by the system having before been partially affected by small-pox or vaccination. Conformably to this hypothesis, which Professor Thompson, as we have seen, has espoused, all these affections, including the varioloid, have one common parent in small-pox, and though somewhat dissimilar in aspect, and other qualities, retain enough of general resemblance to betray their consanguinity or relationship. As the Sea Nymphs of whom Ovid says:

> "Facies non omnibus una,

> Nec divina tamen, qualem decet esse sororem."

Their faces are not the same, though so much alike they might be known to be sisters. Granting, however, all that is contended for in this respect, which I am not prepared to do, it seems to me, every other objection aside, that the repetitions of small-pox have recently so far exceeded all former experience, as to be utterly irreconcilable with the explanation now attempted. Consult the whole history of the disease, and no parallel instance can be found, though it has prevailed epidemically at different times, in a shape quite as formidable as ever known.

By others it has been held that the epidemic in question, was really a malignant or highly aggravated state of varicella. It is admitted, though generally there is no difficulty in distinguishing the two diseases, that occasionally varicella assumes an aspect so imitative of small-pox, as readily to be confounded with it. More than once, it has sporadically appeared, in this city, in such a guise; and we are informed by Willan, (who, I repeat, of all men, was the most conversant with the diagnosis of this order of affections,) that in six years he saw seventy-five cases of chicken-pock, which had been mistaken for small-pox. The resemblance, indeed, is occasionally so close, that, as before mentioned, till within the last half century, they were considered as the same disease, the latter being somewhat modified.

Epidemic varicella, too, has occurred at various times and with so much malignity, as to be mistaken for small-pox, of which a remarkable example is recorded of Sims, of London. As regards our recent epidemic it is indisputably true, that on its original appearance, the prelusive cases of it were decidedly of a varicellous nature. These were in summer, and it is not utterly unreasonable to surmise, that on the accession of cold weather, by the concentration of the contagion, the disease may have been gradually exacerbated into the malignancy which it ultimately assumed. Moreover, neither variolation nor vaccination affords any security against the attacks of varicella, in which particular it corresponds to a certain extent with the epidemic in this city. Nevertheless, the hypothesis is met by such insuperable objections, that I think it must be abandoned,—and especially by the fact, that varicella cannot be propagated by inoculation, in this respect differing from the epidemic in its unmodified state.

Taking all the circumstances into view, I am half inclined, though not absolutely willing, to adopt the opinion, which alleges that this recent epidemic, if not some other disease, was a very essentially altered state of small-pox. The conjecture is, at least, rendered plausible by the consideration of the impossibility of reconciling the multiplied failures which have taken place, as well in vaccination as variolation, with all preceding experience of the infallibility, or nearly so, of these two processes. What becomes, on any other supposition, of the evidence deduced from the infinity of experiments in the early stage of vaccination, for the purpose of determining its efficacy? Numerous individuals, we are told, were exposed, after having gone through this operation, to the most concentrated contagion of small-pox, in hospitals and elsewhere, with entire impunity. These experiments were made, to a very great extent, in London, Paris, Vienna, and this city. It was the practice, too, for a series of years, to subject all cases of vaccination to variolation, as a criterion of the efficacy of the former, and the result of these multiplied trials was as I have stated. No point was seemingly better established, than that an individual having had vaccination was rendered for ever unsusceptible to the action of small-pox. But now, the fact is otherwise, or that this expedient affords a very precarious protection. It has been urged, I am aware, in explanation of these failures, that small-pox, when vaccination was introduced, and its efficacy tested, so far from being of the envenomed character it subsequently assumed, had become so remarkably mild and benignant, that its contagion possessed little force. The suggestion would be entitled to more attention, did not the same degree of fallibility in the process continue to the present moment, when the epidemic has lost all its intensity. Conceding, however, its plausibility in regard to vaccination, I do not see how it can apply to variolation. To the period of which I am speaking, small-pox once thoroughly acquired in this mode or naturally, little or no solicitude was entertained of any future recurrence of it. Not to cite authorities superfluously on this point, let us take a single one, from countries where the subject was probably best understood. The estimate of Heberden in England, of secondary small-pox, was one in five thousand,—of Condamine in France, of one in double this number; and the late Professor Kuhn, of this University, informed me, that in a practice of fifty years, he had never met with a single instance of such an occurrence. But now, the lengthened and accumulated evidence, which sustained its efficacy, is gone, and we are released from a creed, which, slowly and cautiously adopted, was cherished with idle credulity for more than a century. Difficult as it may be to suppose, that we were labouring all this time under so gross a deception, it unavoidably follows, should the disease prove to be genuine small-pox. But, presuming the contrary, or that some new, or exasperated, or otherwise altered old eruption, with the general variolous aspect, though in some particulars of a different character, has appeared, we are supplied with an infinitely more satisfactory solution of this problem.

Nor do I perceive, why we should refuse our assent to this hypothesis. Change is as incident to some diseases as any thing else, and while many are gradually modified, or wholly extinguished, others are suddenly brought into existence. The venereal distemper is an exceedingly pertinent example. Developed by a fortuitous combination of causes, it has, in the progress of time, undergone a most striking revolution in its character and treatment. From the early writers on the subject it may be learnt, that when that disease first appeared, it had the character of a general febrile eruption, propagated by an infectious effluvium, and not particularly by sexual intercourse, or confined in its primary aggression to the genital organs, as in later times.

Nothing more extraordinary is there in the varioloid, than the syphiloid affections. That inoculation should no longer afford absolute security against this modified contagion, is not more surprising, than the inefficiency of mercury in most of the modern forms of syphilis.

Dismissing this part of the inquiry, it may be remarked as very curious, that a similar conjecture was some years ago thrown out by Southey the poet. In a dialogue purporting to be between Sir Thomas More, who lived in the reign of Henry the Eighth, and a personage of the present day, the latter is rebuked for too arrogantly asserting the superiority of modern discoveries and improvements, and especially in relation to small-pox. "What," says More, "if small-pox, which was vainly supposed to be subdued, should assume a new and more formidable character, and, as there seems ground for apprehension, instead of our being protected by vaccination from its danger, it should be ascertained, that inoculation itself affords no security?" This, in some degree, I fear has proved the language of prophecy.

Determining from my own observations, I should say, that of any given number of individuals, however perfectly vaccinated, who might be exposed to the concentrated infection of small-pox, not one-fourth would escape the disease, in some shape or degree,—and in this estimate, I am entirely supported by several of my medical friends. Looking on the data which have been presented in the preceding part of the discussion, it must be deduced, whatever may be our reluctance to do it, that vaccination as a preventive of the epidemic among us, has proved so inefficient as scarcely to deserve to be considered at all in this light.

In Europe the same distrust of the security furnished by vaccination at present exists, as appears from many of the recent publications, and more particularly from Professor Thompson's work on small-pox.

Nor is the testimony of Gregory less decisive; and, from his position as physician to a large vaccine establishment in London, it is particularly authoritative. Though wedded still to vaccination, he candidly confesses, that its failures in the prevention of small-pox, have been steadily on the increase for some years past, and that small-pox after vaccination, is far more frequent, than instances of secondary small-pox. From the register kept in the hospital, we learn, that "in the year 1813, the proportion of cases of small-pox succeeding vaccination, to the whole number of admissions, was as one in thirty—in 1815, as one in seventeen—in 1819, as one in six—in 1821, as one in four—and during the year 1822, as one in three and a half." The subsequent proportion, in that institution or elsewhere, I am without the means of determining. But it seems to be the creed into which the medical mind every where is fast settling down, that vaccination is chiefly valuable as preserving life, by tempering the violence of small-pox. Even thus limited, its utility is great, as will appear more conspicuously, when the fact is proclaimed, that more than one-half, according to pretty accurate reports, died at first, in this city, of the epidemic, in the unprotected system, and not more than one in the thousand, where vaccination had been properly received.

(To be continued.)

